# Turning over New Leaves

**Published:** 1890-03-22

**Authors:** 


### TURNING OVER NEW LEAVES.

### "The Review of Reviews," March, 1890.

THE March number of Mr. Stead's latest editorial venture contains a very large quantity of eminently readable matter upon many subjects. "Greetings from near and far" come from various personages of note, and the assertion that "the first volume of the *Review of Reviews* promises to be unequalled as a collection of autographs of contemporary celebrities," seems likely to be fulfilled. But this, we imagine, is not the ultimate object of the new periodical, and one grave charge at least may fairly be brought against it. In its character of supreme critic and final court of appeal, the *Review of Reviews* ought assuredly to be absolutely impartial and politically unbiassed. Yet if there is one thing particularly noticeable about this number, it is its political animus, no matter in which direction it tends. Any such tendency is wrong according to the notion conveyed by the pretentious title adopted. In practice it must prove fatal to its prosperity, and we hope absolute neutrality in politics will be displayed in future. Probably the most useful feature in the Review is its classified list of recent publications, and the brief synopsis it furnishes of the contemporary magazines at home and abroad. Such a synopsis is extremely valuable when presented in a practicable form. It is an exceedingly difficult thing to do well, and praise is due here for the really clever workmanship displayed. The skeleton novel is not altogether a happy idea, and we should like to hear the opinions of the various authors as to the propriety of having their works denuded of all literary vesture and artistic adornment, that the "story" may be nakedly presented to the world, and deprived of all power to charm. Surely to satisfy the curiosity of the reading public by a succinct statement of the events narrated in a book is to spoil the chances of sale. If sanctioned, it is unkind; if unsanctioned, it is very like literary piracy. No man of judgment, however, after examining the current number of the *Review of Reviews* will be able to deny that it shows great editorial ability, much administrative grasp, and a wisdom of selection that has seldom, if ever, been excelled. Time alone will prove if this review will adequately fill the very high position it aspires to occupy.

